# Editorial: Using Motion Analysis Techniques and Musculoskeletal Modeling of the Spine to Better Understand Spinal Disorders and Evaluate Treatment Effects

**DOI:** 10.3389/fbioe.2022.884123

**Published:** 2022-04-06

**Authors:** Dennis E. Anderson, Stefan Schmid, Lennart Scheys, Babak Bazrgari

**Affiliations:** ^1^ Beth Israel Deaconess Medical Center, Boston, MA, United States; ^2^ Harvard Medical School, Boston, MA, United States; ^3^ School of Health Professions, Spinal Movement Biomechanics Group, Bern University of Applied Sciences, Bern, Switzerland; ^4^ Faculty of Medicine, University of Basel, Basel, Switzerland; ^5^ Department of Development and Regeneration, Faculty of Medicine, Institute for Orthopaedic Research and Training (IORT), KU Leuven, Leuven, Belgium; ^6^ F. Joseph Halcomb III, M.D. Department of Biomedical Engineering, University of Kentucky, Lexington, KY, United States

**Keywords:** motion capture, multi-body modeling, electromyography, spinal pathology, back pain, treatment intervention, prevention

## Background of the Research Topic

The pathomechanics of many musculoskeletal spinal disorders are still poorly understood, which makes it challenging to develop and apply effective preventive strategies, treatment modalities and rehabilitation plans (Joshi et al., 2019; Taşkıran, 2020). Moreover, the outcomes of currently practiced care pathways are usually evaluated through clinical tests, medical imaging, or questionnaires, whereas the effects on the functional dynamics of the spine remain largely unknown (Matthew et al., 2018; Severijns et al., 2020). For these reasons, this Research Topic aimed at reporting recent efforts to apply key biomechanical analysis techniques, specifically motion analysis and musculoskeletal modeling, to study spinal disorders and their treatments. With advances over the past decade in the fields of motion capture and detailed musculoskeletal modeling of the spine, these approaches have clear potential to provide new insights into onset and progression of spinal disorders, as well as the effects of current treatments, on the biomechanical function of the spine.

To provide a complementary forum for discussion among interested researchers, we organized a virtual symposium and invited interested authors to participate. The symposium was held on 12 May 2021, with a total of 13 presentations on studies in this area from authors of nine different countries and a total of 130 participants from over 20 different countries. The abstracts and videos from this symposium may be found online here: *Motion Analysis and Musculoskeletal Modeling in Treatment of Spinal Disorders* .

## Overview of the Research Topic Collection

The Research Topic has a total of 13 papers, including methodological developments in spine motion analysis and modeling, studies of how spinal disorders affect spine biomechanics, and studies of treatments and interventions. Measurement of spinal motion was a component of eight studies presented here, including six using optoelectronic motion capture, one using inertial measurement units (IMUs), and one using quantitative video fluoroscopy. Three studies used biplanar radiography (the EOS imaging system) to evaluate 3D spinal posture or curvature. Four studies used musculoskeletal models to evaluate spinal loading, and one study used finite element models to evaluate spinal strain. Seven studies examined specific spinal disorders with measurements in patients, including chronic low back pain, lumbar spinal stenosis, adolescent idiopathic scoliosis, and adult spinal deformity. Four studies focused on the effects of treatments, and two on the use of biomechanical loading outcomes to inform prediction or prevention of spine conditions.

## Developments in Motion Analysis and Modeling

Advances in the state of the art for motion capture and musculoskeletal modeling have enabled new studies focused on spinal disorders in recent years, as evidenced by many of the studies in this Research Topic. However, efforts continue to develop and improve these methods. Severijns et al. quantified palpation error on the positioning of spinal markers, and how it would affect estimates of spinal postures. Mediolateral palpation error was larger in adult spinal deformity (ASD) patients than healthy controls, but other errors were similar, and correcting palpation errors improved marker-based estimates of spine curvature. Overbergh et al. examined the test-retest reliability of spinal kinematics evaluated during seated forward bends in ASD patients based on subject-specific kinematic models. Excellent reliability was reported for some range of motion (ROM) outcomes (e.g., lumbar lordosis), but poor for others (e.g., thoracic kyphosis and pelvic tilt), and test-retest variability was greater than operator-induced uncertainty. Fasser et al. presented a procedure for generating subject-specific musculoskeletal models based on bi-planar radiography data (EOS imaging system), using these to set skeletal geometry as well as mass distributions. Breen et al. reported on lumbar intervertebral motions in healthy subjects during flexion and return motions, using quantitative video fluoroscopy. This provides an important reference standard for spinal motion, which is needed for validating other approaches for spinal motion analysis and for future comparison in patients with spinal disorders.

## Spinal Disorders Affect Spine Motion and Loading

Several studies reported significant effects of spine disorders on spinal motion or loading. Saad et al. showed that patients with ASD, particularly those with sagittal or hyperkyphotic deformities, adopt different movement strategies than healthy controls during sit-to-stand motions, including maintaining a flexed trunk and more sagittal trunk ROM. Similarly, Christe et al. examined various functional spinal movements in patients with chronic low back pain and found that sagittal movement patterns can distinguish patients from healthy controls, particularly angular velocities which are lower in patients. Finally, Mousavi et al. examined walking in patients with lumbar spinal stenosis. The presence of neurogenic claudication symptoms minimally affected spine motion and posture, but increased estimated lumbar spine loading by 7%.

## Evaluations of Potential Clinical or Preventive Applications

Two studies examined spine biomechanics in patients with adolescent idiopathic scoliosis, but with different goals. Kinel et al. assessed the ability of patients to perceive and self-correct their posture and 3D spine shape. Most patients were unable to improve their spine shape instinctively, pointing to the need for careful training in appropriate exercises to develop this capacity. Bassani et al. examined the use of biomechanical parameters, including muscle and intervertebral forces, to predict scoliotic curve progression of adolescent idiopathic scoliosis. However, accounting for these measures did not improve the prediction. Huang et al. used motion analysis to quantify the motion during two different cervical manipulations (in healthy people). Two studies included simulations of spinal fusions: Loenen et al. examined the effect of a titanium interbody cage for spinal fusions on strain patterns in patient-specific finite element models, while Rasmussen et al. performed a simulation study of how lumbar fusions affected spinal loading using musculoskeletal models. The latter suggests that lateral or asymmetric lifts are a particular concern, with post-operative increases in loading regardless of surgical approach. Finally, and relevant to prevention of spinal disorders, von Arx et al. used motion capture-driven musculoskeletal full-body models to investigate spinal loads under three types of lifting techniques. Stoop lifting thereby resulted in lower compressive loading compared to squat and freestyle lifting. And even though stoop lifting resulted in higher anterior-posterior shear forces in the mid to upper parts of the lumbar spine, they were the lowest in the L5/S1 segment, which is most frequently affected by degenerative spinal disorders.

## Conclusion

Collectively, the studies presented in this Research Topic used motion analysis and musculoskeletal modeling to gain new insights into spinal disorders and highlight the potential of these methods for clinical applications. The methodological development studies provide important information on the current state-of-the-art in motion analysis and modeling of the spine and help address limitations that must be overcome for widespread clinical translation of these techniques. The studies of spinal conditions demonstrate that the biomechanical effects of various spinal conditions are detectable using current biomechanical analysis methods, supporting future efforts to elucidate these effects and translate these methods to clinical applications. Finally, several studies here provide important examples of how biomechanical analyses may be helpful in planning, developing, or optimizing treatments, as well as preventive strategies. As editors of, and contributors to, this topic, we sincerely hope this collection of studies can serve as a roadmap to accelerate translation of motion analysis and modeling to clinical management of spinal disorders and can ultimately lead to design and development of new and more effective interventions for spinal disorders. We look forward to continued efforts over the next few years to leverage the methodological developments and examples found here toward clinical applications in this exciting and emerging field.
